# AlphaFold2 Reveals Structural Patterns of Seasonal Haplotype Diversification in SARS-CoV-2 Spike Protein Variants

**DOI:** 10.3390/biology13030134

**Published:** 2024-02-21

**Authors:** Muhammad Asif Ali, Gustavo Caetano-Anollés

**Affiliations:** Evolutionary Bioinformatics Laboratory, Department of Crop Sciences, University of Illinois at Urbana-Champaign, Urbana, IL 61801, USA; maa32@illinois.edu

**Keywords:** COVID-19, haplotypes, variant of concern, AlphaFold, spike protein, mutation, protein structure, evolutionary pressure, pandemic, recruitment

## Abstract

**Simple Summary:**

The COVID-19 pandemic showcases the impact of mitigation and elimination strategies across the globe, including the development of effective vaccines, antiviral drugs and diagnostic tools. However, the virus changes rapidly over time. Consequently, control strategies have been limited by time-consuming experimental acquisition of three-dimensional atomic protein structures of the fast-developing mutant ‘variants’ of the virus, which remains an unviable strategy for fast and effective disease control. Here, we use AlphaFold2 to model the atomic structure of the ever-changing SARS-CoV-2 spike protein in silico. AlphaFold2 is an artificial intelligence (AI) deep learning computational tool capable of producing models at experimental resolution in only a few hours. Structural models for major Variants of Concern (Alpha, Delta, and Omicron) and latitude-delimited haplotypes, sets of genetically linked and highly prevalent mutations that impact the epidemic calendar of the virus, were compared to the structure of the reference Wuhan strain. We find that patterns of structural change triggered by seasonal haplotype diversification could help predict the changing face of the virus, understand seasonal behavior, and develop more resilient vaccines and drugs.

**Abstract:**

The slow experimental acquisition of high-quality atomic structures of the rapidly changing proteins of the COVID-19 virus challenges vaccine and therapeutic drug development efforts. Fortunately, deep learning tools such as AlphaFold2 can quickly generate reliable models of atomic structure at experimental resolution. Current modeling studies have focused solely on definitions of mutant constellations of Variants of Concern (VOCs), leaving out the impact of haplotypes on protein structure. Here, we conduct a thorough comparative structural analysis of S-proteins belonging to major VOCs and corresponding latitude-delimited haplotypes that affect viral seasonal behavior. Our approach identified molecular regions of importance as well as patterns of structural recruitment. The S1 subunit hosted the majority of structural changes, especially those involving the N-terminal domain (NTD) and the receptor-binding domain (RBD). In particular, structural changes in the NTD were much greater than just translations in three-dimensional space, altering the sub-structures to greater extents. We also revealed a notable pattern of structural recruitment with the early VOCs Alpha and Delta behaving antagonistically by suppressing regions of structural change introduced by their corresponding haplotypes, and the current VOC Omicron behaving synergistically by amplifying or collecting structural change. Remarkably, haplotypes altering the galectin-like structure of the NTD were major contributors to seasonal behavior, supporting its putative environmental-sensing role. Our results provide an extensive view of the evolutionary landscape of the S-protein across the COVID-19 pandemic. This view will help predict important regions of structural change in future variants and haplotypes for more efficient vaccine and drug development.

## 1. Introduction

In December of 2019, an outbreak of pneumonia erupted in the city of Wuhan, Central China. The culprit was a novel coronavirus believed to have originated from bats. The virus was subsequently named Severe Acute Respiratory Syndrome Coronavirus 2 (SARS-CoV-2) and the disease COVID-19 [1,2]. COVID-19 caused a multiplicity of symptoms, including high fever, headaches, consistent dry coughing, severe breathing difficulty, and pneumonia [2]. Prolonged disease often resulted in failure of the respiratory system due to alveolar damage (requiring life support from a mechanical ventilator) or even death. The disease quickly spread globally and currently persists as an ongoing pandemic. As of 31 December 2023, there have been 6,991,842 total COVID-19 deaths reported by the WHO (https://covid19.who.int/, accessed on 31 December 2023). This pandemic represents the seventh instance of a coronavirus outbreak in humans in just the past 20 years [3], with the previous two instances being SARS (March 2003, Guangdong, China) and MERS (September 2012, Jeddah, Saudi Arabia) [4,5].

SARS-CoV-2 shares ~96% of the sequence of its genome with RaGT13 (a SARS coronavirus infecting the horseshoe bat) but shows significant differences in the spike (S) protein, especially in the receptor-binding domain (RBD) region of the molecule [1]. These differences explain its higher affinity to the Angiotensin-Converting Enzyme 2 (ACE2) receptor, the central disease entry point that is present in lung tissues, which increases pathogenicity. The transmembrane protease serine 2 (TMPRSS2) enzyme helps activate the S-protein for binding to the host cell membrane [6,7]. The S-protein plays a vital role in viral pathogenicity, which is why current and past efforts have focused on targeting the S-protein and its interacting molecules for the development of vaccines, therapeutic medicines, and diagnostic tools. However, such efforts are being constantly renewed due to the rapid evolution of RNA viruses [8], with SARS-CoV2 mutating at a rate of 1.3 (±0.2) × 10^−6^ mutations per-base per-infection cycle [9]. Currently, eight variants of Omicron, the current ‘Variant of Concern’ (VOC) (first reported by the Centers for Disease Control (CDC) in the US on 27 October 2022), are being monitored (https://www.cdc.gov/coronavirus/2019-ncov/variants/variant-classifications.html, accessed on 31 December 2023). The wild-type (WT) Wuhan strain, the first instance known of SARS-CoV-2, exhibited lower levels of phenotypic adaptation in humans, which is in stark contrast to its later variants [10]. This is expected, as the virus is most likely the result of natural selection after zoonotic transfer [11]. This process requires the virus to adapt by selecting the best mutations impacting the structure and function of viral proteins [12]. The main VOCs replaced one another by increasing virus fitness in the context of T cells, innate immunity and population immunity, and a complicated relationship between virus antigenicity, transmission, and virulence [13].

Three VOCs, Alpha, Delta, and Omicron, were the most widespread across the globe, with later variants completely replacing earlier variants in a very dynamic evolutionary landscape of mutation accumulations [14]. Each of these variants brought about their own sets of mutations, with VOC Omicron being increasingly concerning as mutations tended to be inherited across variants. VOCs Alpha and Delta were accompanied by 9–12 mutations along the S-protein, while VOC Omicron initially expressed a total of 30 mutations [15]. Mutations impacted the S-protein differently depending on specific sites along the S-protein but also in the degree of change brought to said regions, showing a continuum of change where the virus seems to explore the best possible structural conformations to enhance function and survival. The constant adaptation of SARS-CoV-2 to its new human host is causing vaccine, drug, and diagnostic development efforts to slow down due to the time required to obtain high-quality atomic structures of proteins using, for example, cryogenic electron microscopy (cryo-EM). A few months after Walls et al. [16] made three-dimensional (3D) structural models of the spike available in March 2020, VOC Alpha emerged in the UK and spread globally due to its increased transmissibility [17]. This presented a challenge against vaccination efforts, as the virus kept changing at a pace faster than the acquisition of high-quality 3D models of the viral proteins.

With the advent of artificial intelligence (AI) tools such as AlphaFold2 [18], the winner of the CASP13 and CASP14 structural prediction competitions, structural biologists are now able to produce near-experimental resolution 3D models for most proteins. The latest AlphaFold2 predictions have a median backbone accuracy of 0.96 Å r.m.s.d.95, whereas the next best model has an accuracy of 2.8 Å r.m.s.d.95 [18]. AlphaFold2 now provides a much faster alternative to obtaining 3D models than cryo-EM, taking up only a few hours of computation. The tool can hence be used to model sequences of the SARS-CoV-2 variants as they appear and have their structures analyzed. In a recent study, for example, an AlphaFold2 modeling approach was used to screen drug targets against numerous variants ranging from VOCs Alpha to Mu, highlighting the differences among each of them [19]. Structural models were validated against the cryo-EM structure of the Wuhan strain. The study found numerous regions of structural change, especially across the N-terminal domain (NTD) and RBD regions in the S1 subunit of the S-protein. When compared to all other VOCs, VOC Delta experienced the most structural change. In an earlier study, Heo and Feig [20] applied a similar approach to model four non-structural proteins (NSPs), five ORF-defined proteins, and the membrane (M) protein, but used both AlphaFold and C-I-TASSER along with improved maximum likelihood (ML) and physics-based refinements to improve structure reliability. AlphaFold2 structures for the SARS-CoV-2 protein repertoire have been included in the PDB data repository [21] as they provide data for comparative and functional analysis but also highlight the importance and reliability of having AlphaFold2 structures for research [22]. The use of AlphaFold2 modelled structures for structural analysis of variants has also been utilized to guide vaccination strategies, showing the number of doses required for the effective neutralization of a variant [23].

VOCs, however, do not provide a complete picture of the viral mutational landscape. While individual periods of the pandemic associate with VOCs and their mutation constellations, VOCs are highly dynamic and a collective of other mutations co-exist with their mutational makeup. These other mutations can be more prevalent than mutations in VOC constellations among the population of sampled sequences. This presents a problem of combinatorics. In this regard, the work of Tomaszewski et al. [14] lays the foundation for modeling the most relevant sets of mutations associated with virus seasonal behavior making up VOC constellations in the form of haplotypes. Haplotypes are sets of mutations (genetic signatures) that tend to be linked (inherited) together in viral variants. In this study, the emphasis is put on the three major VOCs, Alpha, Delta, and Omicron, along with the individual seasonal haplotypes defined by latitude and temperature (which are tightly correlated) that make up each of their mutant constellations, from haplotype 1 (H1) to haplotype 17 (H17). The rationale for focusing on major VOCs is that most of the other variants were not as prevalent globally and were quickly replaced by the much more dominant VOCs Alpha, Delta, and Omicron. Additionally, haplotype recruitment and coalescence played a crucial role in the survival/fitness of developing variants, which necessitated the modeling of individual haplotypes. The co-existence of these haplotype sets within VOCs likely provides an evolutionary benefit to the virus by acting as a buffer against mutations that negatively impact virus survival under different climatic conditions. Haplotype interactions could also aid infection as combinatorics of different types of viral particles could help evade host immune responses.

The available AlphaFold2 models used in the previously mentioned studies [19] only model the SARS-CoV-2 proteins with variant definitions (VOC constellations). However, this approach loses information on the patterns and trends of haplotype coalescence and decoupling that occurred during the course of the COVID-19 pandemic [14]. Haplotypes can originate independently but are later recruited into variants to become larger stable haplotypes in a process of coalescence. However, they can also recruit prior haplotypes or develop sets of mutations that distinguish themselves by their global accumulation patterns, causing mutations to decouple from their originating variant [14]. Since the different haplotypes have mutations located in different regions of the S-protein, each haplotype will impact molecular structure differently. Here, we further dissect these patterns of haplotype interaction and provide new information into the evolution of the structure of the S-protein as the virus continues to fit changes in protein sequence to those of 3D structure. We show that some protein regions/domains exhibit little to no structural deviation, an observation that indicates higher regional stability. These regions that are more stable and less likely to change over time could be exploited in mitigation efforts, with the hope they would minimize immune evasion caused by structural and conformational changes in the protein and make mitigation effective for longer periods of time. We also report that the NTD region of the S-protein exhibited the highest number of structural changes, especially in VOC Omicron, and that the RBD also experienced huge structural shifts involving translations and twisting away of sorts in 3D space, especially involving a cavity region that binds antibody C144 and has neutralizing potential [24]. Since the NTD has been shown to be linked to seasonal behavior [25] and the RBD is the most functional part of the S-protein in human (h)ACE2-binding activity, our findings are of great significance. We also show that the S1/S2 boundary known to carry a cleavage site necessary for post-binding membrane fusion activity [26] is a region of high structural change. Interestingly, our structural analysis reveals regional patterns where the virus tends to undo some extreme effects from earlier VOCs/haplotypes, similar to how the virus experiences entropy fixation and reversals with respect to mutations at different sites [25,27].

## 2. Materials and Methods

### 2.1. AlphaFold2 Modeling

The structure of the SARS-CoV-2 S-protein encoded in the genomic sequence of the Wuhan strain (the WT) was compared to the structures of mutated variants, VOCs Alpha, Delta, and Omicron, along with their accompanying haplotypes. The Wuhan reference fasta sequence (EPI_ISL_402124) was obtained from the online web service GISAID [28]. VOC and haplotype definitions were retrieved from the data curated by Tomaszewski et al. [14]. Table S1 describes the order of progression of VOCs and haplotypes and their associated mutations. Local ColabFold, an offline version of AlphaFold2 [29], was used to model each of the proteins using a computer workstation with the following specifications: AMD Ryzen Threadripper 2990WX 3.0 GHz 32-Cores, 128GB Ram, NVIDIA GeForce RTX 2080 Ti Rev.A, running Ubuntu 22.04.1 LTS 64-bit version. The best 5 models were ranked using their pLDDT scores, which is AlphaFold’s metric of choice for evaluating the quality/confidence level of a structure. Structural alignments of lower-ranked models to rank 1 models and the modeled Wuhan reference structure (Table S2) revealed that the RMSD values of rank 2 models were similar to the rank 1 structures with each VOC and haplotype showing 1100+ amino acid residues falling under 1 Å. In contrast, less than 350 residues of the rank 3, 4, and 5 models had RMSD values of ~1 Å. We used the relaxed rank 1 versions in further structural comparisons given the lower pLDDT scores and poor structural alignments of lower-ranking models. AlphaFold2 produces a confidence estimate for each residue from a scale of 1 to 100, which can be used to make inferences on the quality of a structure produced. Overall pLDDT scores per structure were also produced in the output [30]. The top-ranked model of the S-protein had scores above 82 for the Wuhan, Alpha, Delta, and Omicron strains, which is considered exceptionally good as scores within the range of 70–90 have very accurate backbone predictions [30]. With most structures achieving high pLDDT scores of around 80 and the S-protein being predominantly ordered with very few regions approaching intrinsic disorder levels [27], the structures are reliable for use in comparative analyses.

### 2.2. Structural Analysis

Chimera [31] was used to visualize protein structure and to identify regions of structural deviation. All structures were superimposed onto the Wuhan reference, and individual header files containing the per residue RMSD scores were extracted to record regions of structural deviation based on RMSD thresholds of 2, 3, and 5 Å. Regions of high deviation crossing 3.0 Å were then further analyzed using USalign [32] to score the regions of structural change across each VOC and haplotype.

### 2.3. Validating the AlphaFold2 Models

For validation purposes, we used the AS2S [33], US-align [32], and TM-Score [34] online servers to evaluate the accuracy of the AlphaFold 2 models against cryo-EM models. The AS2S server allows us to calculate the Global Distance Test Template Score (GDT-TS) implemented in the Local Global Alignment Method, which represents a much better metric of protein similarity than the RMSD metric, which is heavily influenced by small changes [33]. The GDT-TS circumvents all major issues. US-align was used to measure regional Template Modeling (TM) scores of regions identified as structurally different from the Wuhan strain, whereas the TM-Score server was used to visualize what portion of the AlphaFold2-modeled S-protein falls within 5 Å of a cryo-EM model.

## 3. Results

### 3.1. Full-Length S-Protein Analysis Using Backbone RMSD

**Figure 1:**
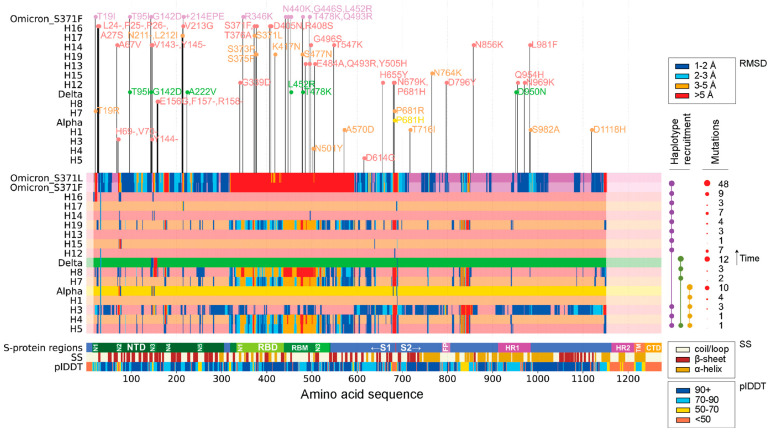
Regions of structural deviation (measured in RMSD). The vertical axis represents each of the haplotypes and variants discussed in this paper arranged in chronological order from earliest to latest, up until the appearance of VOC Omicron. The horizontal axis represents the amino acid positions along the length of each of the S-protein molecules. The horizontal bars representing the haplotypes and VOC constellations are colored in hues of light coral and sandy brown in an alternating fashion, whereas VOCs Alpha, Delta, and Omicron are colored yellow, green, and purple, respectively. When a residue along the length of any of these protein molecules crosses the 1 Å, 2 Å, 3 Å, and 5 Å RMSD, then that position is colored in dark blue, light blue, orange, and red, respectively. The horizontal bar labeled ‘S-protein regions’ indicates all positions of important regions/domains along the S-protein. The horizontal bar labeled SS indicates the position of alpha helices (brown), beta sheets (dark red), and coils (beige). The horizontal bar labeled plDDT represents the confidence level of AlphaFold2 for each residue along the S-protein. On top of the graph are the individual mutations that make up each of the haplotypes and variants. Note that free-standing mutations are not modeled independently but are included and marked in the variant on this graph. Table S1 lists VOCs and haplotypes and their associated mutations.

Figure 1 visualizes the amount of structural change in the S-protein brought about by the appearance of each VOC and corresponding haplotypes during the duration of the SARS-CoV-2 pandemic. Individual structural alignments were used to gather the per residue RMSD of each molecule, providing a holistic view of which areas of the S-protein experienced the least to the most amount of structural change. When using an RMSD threshold of 3 Å, the S1 subunit seemed to host many of the structural changes that developed during the pandemic. In contrast, the S2 subunit remained relatively unchanged, as did the fusion protein (FP) site and the region spanning the FP and the heptad repeat 1 (HR1) regions. Note that the S1 region is known to form the bulbous head while S2 forms the stalk region of the S-protein [35]. Structural deviations coincided with functionally relevant segments of the protein, including the RBD and the N1-N5 loops of the NTD regions of the S1 subunit. These deviations manifested throughout the pandemic. The S1/S2 boundary also exhibited significant change. Interestingly, many of these regions were mainly comprised of loops and coils. The structural change experienced by the S-protein at different phases of the pandemic was not uniform, with some VOCs and haplotypes experiencing more change than others. When following the order of appearance of VOCs and haplotypes, the majority of changes were introduced by VOC Omicron and haplotypes H5, H4, H3, H7, H8, and H19. Mutation numbers also failed to indicate whether a VOC or haplotype had more or less structural deviation from the original Wuhan structure. VOCs Alpha and Delta showed much greater numbers of mutations than their constituent haplotypes but experienced very little change in areas other than the NTD region, including the N2 and N3 loops. The RBD revealed very defined phases of structural change following the appearance of VOC Omicron and haplotypes H5, H4, H7, H8, and H19, but showed little to no structural deviation between phases. The S1/S2 boundary observed the same pattern with an exception in haplotype H3. Other studies utilizing AlphaFold2 for structural comparisons of VOCs with the Wuhan strain also report notable changes at the NTD supersite and the RBM subdomain [19,36]. 

Consistently, the N2 and N3 loops of the NTD were the most impacted areas of the S-protein throughout the pandemic. The conformation of the N2 loop, however, was not significantly altered in VOC Delta and corresponding haplotypes but was later affected by VOC Omicron and H15. Another region of interest spanned the N3 and N4 loops of the NTD, which showed significant structural change in haplotypes H3, H4, H8, and H19. In VOC Omicron, this region, along with the N4 loop, formed a much larger segment of structural deviation. This behavior suggests that VOCs can have either synergistic or antagonistic effects on the structure of the S-protein. In fact, VOC Omicron behaved synergistically by expanding the region of structural change, while VOCs Alpha and Delta behaved antagonistically, undoing the effects of their individual haplotypes. 

These synergistic and antagonistic effects were most evident in the structural deviation experienced by the RBD across all three VOCs and corresponding haplotypes. VOCs Alpha and Delta acted antagonistically to the structural changes brought about by their haplotypes. H4 and H5 showed high structural deviation, with a few small stretches of residues crossing the 5 Å threshold at all three RBD sub-regions, N1-N3. However, the structure of VOC Alpha seemed to show almost no change at RMSD thresholds above 1 Å. VOC Delta exhibited a similar behavior, showing almost no change in the RBD, but with H8 expressing much higher levels of structural deviation [see wider red bars especially associated with the receptor-binding motif (RBM) in Figure 1]. In contrast, VOC Omicron behaved synergistically as it amplified the impact of all its haplotypes to affect the RBD much more strongly. VOC Omicron recruited H5, H4, and H19, all of which exhibited significant structural deviation in the RBD region at RMSD thresholds in the 3–5 Å range. This contrasts with VOC Omicron and its structural deviations at thresholds above 5 Å. The S1/S2 boundary and the region between FP and HR1 exhibit similar behavior, with VOCs Alpha and Delta suppressing the impact of their haplotypes and VOC Omicron retaining haplotype change (though reducing the number of impacted residues in between the FP-HR1 region).

### 3.2. Characterization of Types of Structural Deviation

**Figure 2:**
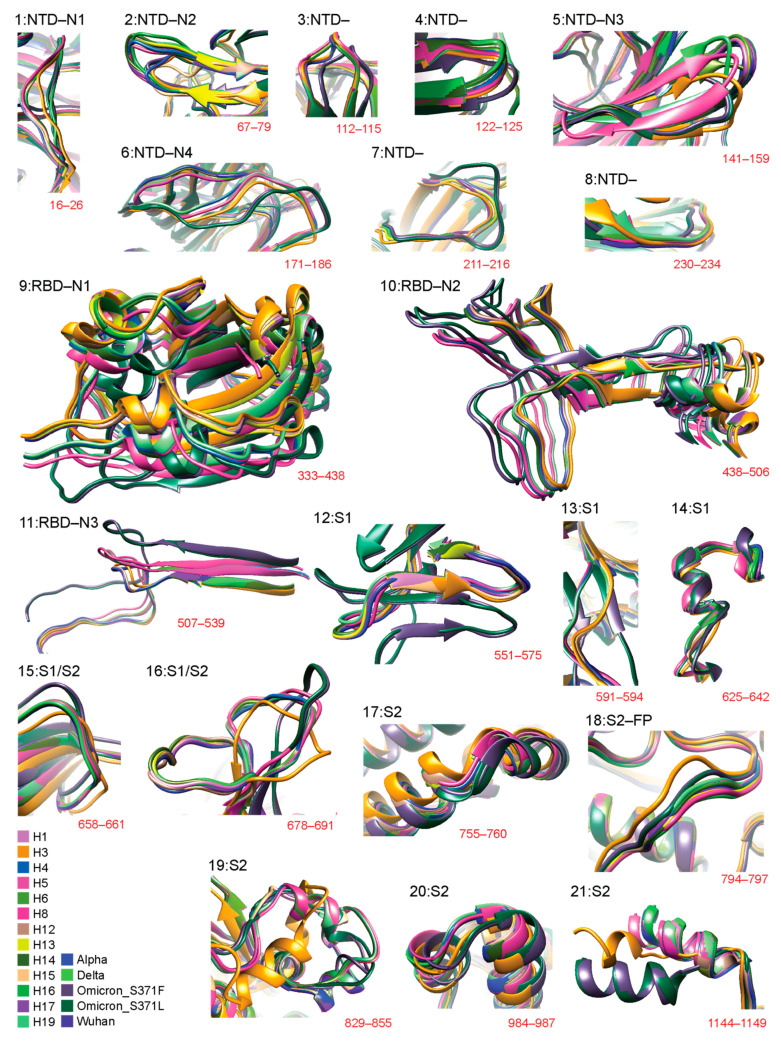
Three-dimensional (3D) models of molecular change at the atomic level in regions of structural deviation. Using the data from Figure 1, all residues that surpassed the 3 Å threshold were translated into 21 regions of structural deviation. Model snapshots of these regions were taken in Chimera to ensure all of the superimposed structures and their corresponding residues were adequately captured for each region. The location of regions in the amino acid sequence is indicated in red. Each variant and haplotype structure is color-coded according to the bottom-left index.

**Figure 3:**
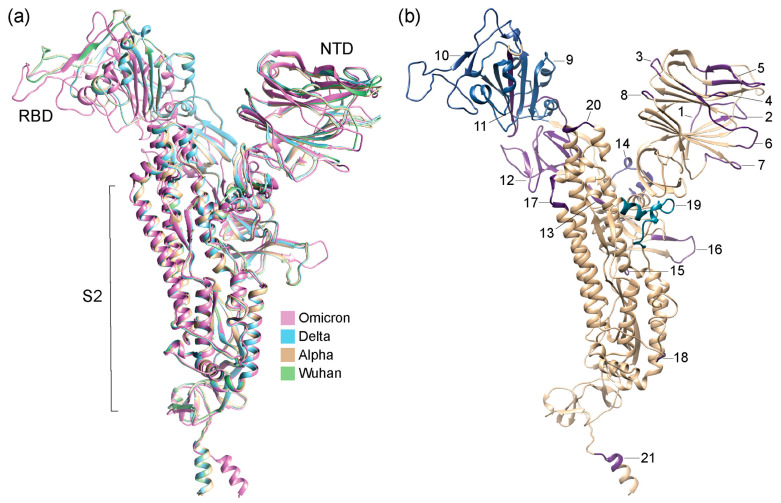
Structural models of S-proteins with regions of structural change (region 1 to 21 as identified in Figure 2) mapped onto them and colored according to TM scores from US-align and Chimera alignments. (**a**) Structural alignment of AlphaFold2-modeled structures of VOCs and Wuhan reference strain. (**b**) Structural model of VOC Omicron. Numbers indicate regions of structural change.

Figure 2 details 3D structural changes in regions of the S-protein identified as significantly impacted throughout the pandemic. The RMSD headers from individual structural alignments were used to pinpoint locations of structural change. Individual comparisons of VOCs and haplotypes against the Wuhan structure are available in Figure S1 (for 12 regions: 2, 5–7, 9–11, 14, 16, 18, and 19). Figure 3 places these changes in the context of the entire molecule. Remarkably, 12 out of 21 of the identified regions of structural change involved loop regions (Figure 2) in the form of either entire loops or loops making up the majority of the deviant structure (regions 1–4, 6–8, 12, 13, 15, 16, and 18). Aside from region 18, all of these loop regions occurred in the S1 subunit and over half of them (7) occurred within the NTD. Loop structures either shortened, lengthened, or experienced significant shifts in 3D space, such as twisting away from the original conformation. Most regions of structural change encompassed small segments of the S-protein. One exception involved a large conformational shift of the entire RBD structure of region 9 (Figure S1) caused by the addition of a short beta-sheet (3 residues in length) at residue 310 in the VOC Omicron molecule. We also detected major changes in the NTD involving the N2 and N3 loops. These changes had effects that were more complex, involving changes in loop length due to deletions or complete changes in regional morphology. Other regions exhibiting similar behavior included region 16, located at the S1/S2 boundary, and region 19, located in the S2 subunit, both of which experienced major conformational changes induced by multiple VOCs and haplotypes.

### 3.3. Regional Analysis with TM Scores Using USalign

**Figure 4:**
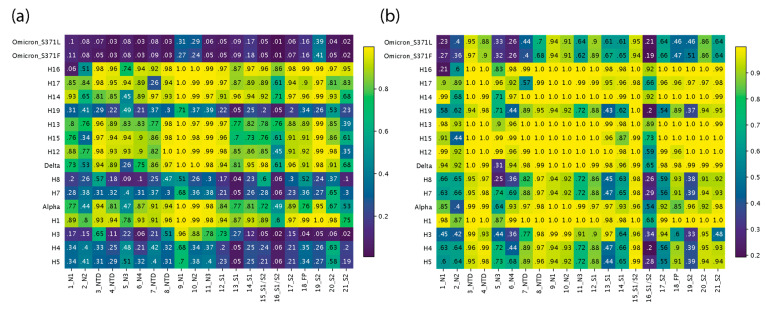
Heatmap of TM scores (ranging from 0 to 1) of regions of structural change. The 21 regions of structural change that we identified were used to slice the corresponding regions from each file and obtain TM scores for each VOC and haplotype with USalign, using the Wuhan reference molecule as the template. The vertical axis is arranged in chronological order, depicting changes across the timeline of the pandemic, and the horizontal axis represents the 21 regions described in Figure 2. All TM scores falling under the TM threshold of 0.5 are colored in white. (**a**) TM scores using alignments from Chimera superimposition. (**b**) TM scores using USalign alignments.

The identified regions of structural change can be better analyzed using TM scores of structural similarity for the group of atoms that are involved. This similarity measure is superior to recording single-residue RMSD changes, which are very sensitive to small changes in conformation. Figure 4 provides heatmap descriptions of per region TM scores collected using alignments from Chimera or USalign. Lighter colors represent high similarity and darker colors have low similarity of structural comparisons. Figure 3 and Figure S2 map regions and TM scores onto S-protein molecules of VOCs.

The analysis revealed four phases of structural exploration in which VOCs and haplotypes experienced significant structural change (e.g., Figure 4a). These phases, which are evident from the dark horizontal bands in the heatmap for VOCs and haplotypes, encompassed time periods spanning the first appearance of H5 and H3, H7 and H8, H19, and the entire mutant constellation of VOC Omicron. There was a continuum of structural change where TM scores kept fluctuating from high to low across VOCs and haplotypes. This fluctuation suggests phases of structural exploration were followed by phases of structural stability. In the periods of stability, the virus experienced structural change in a much more targeted manner where most of the structural deviation was focused on far fewer regions. Both VOCs Alpha and Delta were left out of these phases of exploration despite involving entire mutant constellations, but both saw significant change occurring in the N2 and N3 sites of the NTD and at the S1/S2 boundary (region 16), with TM scores close to or under 0.5. VOC Alpha showed similar scores to prior haplotypes, except for H3, which had a much lower TM score for N2 and N3 at about or below 0.2 and a close to 0 score at the S1/S2 boundary (region 16). VOC Alpha seems to be balancing this extreme impact on the molecular structure and the same can be said for VOC Delta, which is capable of balancing the extreme effects of H8 on the structure at N3 and at the S1/S2 boundary (region 16). These are the same antagonistic behaviors discussed previously. In contrast, the haplotypes of VOC Omicron acted in synergy to amplify the effects of structural change. The N2 and N3 regions of the NTD along with the S1/S2 boundary (region 16) were the only regions that were impacted during phases of stability, with the only exception being region 7 of haplotype H17, which experienced a very low TM score of 0.26 (Figure 4a). Regions 1 and 21 are not discussed here as they occur at the end of the molecules and their conformations could be heavily influenced by low-confidence residue stretches of low plDDT.

Figure 4b describes a heatmap of TM scores generated from alignments implemented with USalign. The approach does not use the original multiple sequence alignment (MSA) generated by Chimera. Instead, it forces a second alignment which often gives higher TM scores for regions of low structural deviation. The approach revealed regions (areas on the heatmap) that experienced greater structural changes (low TM scores), including lengthening or shortening of loop regions and twisting away conformations that entirely departed from the original structure. The heatmap showed that besides the N2 and N3 sites of the NTD and the S1/S2 boundary, other regions also maintained low TM scores. These included the N4 site of the NTD, region 13 of S1, and region 19 of S2. The entirety of the RBD was no longer captured by this method, indicating that the only difference was the shift to the structure caused by the residues at the start of the domain. However, the N3 subregion of the RBD did see lower scores than the other two subregions. This approach helped pinpoint regions of very high structural change because they exhibit drastic structural change captured by crossing TM thresholds after a second regional alignment. Figure S1 describes these regions in greater detail, with an atomic-level view of the differences between corresponding atoms of S-proteins from VOCs and haplotypes.

### 3.4. Significant Structural Changes Suggestive of Recruitments across VOCs and Haplotypes

Table 1 summarizes the significant structural changes that occur across regions of the S-protein with qualitative assessments that are suggestive of trends of structural recruitment throughout the pandemic. The analysis of atomic differences between VOCs and haplotypes for each region reveals recruitment from earlier haplotypes to later haplotypes, especially in H5, H4, H7, H8, and H19, as these adopt similar structures. Also note that VOCs Alpha and Delta only show deviations at the N2 and N3 sites of NTD, both of which are not shared by other VOCs or haplotypes. With the exception of the N2 site of NTD, VOC Omicron adopts unique conformations different from all prior haplotypes.

### 3.5. Benchmarking AlphaFold2 Reference Structures against Experimental Cryo-EM Models

**Figure 5:**
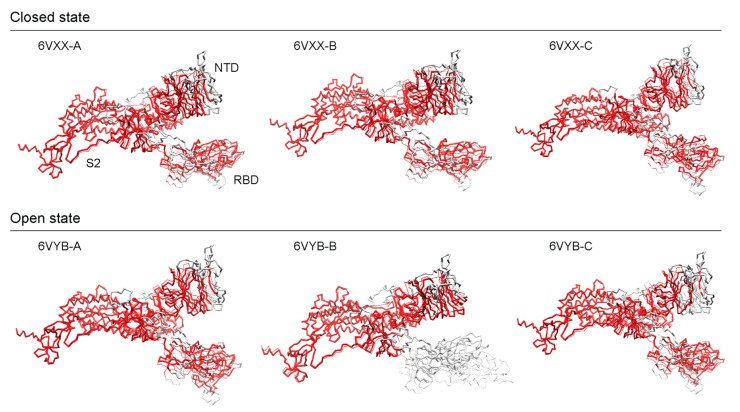
Structural alignments of AlphaFold2 structures against cryo-EM references.

To validate our findings, we compared the AlphaFold2 structures against experimental cryo-EM atomic models of the S-protein [16]. Cryo-EM experiments omitted the Furin cleavage site that spans the S1 and S2 subunits and produced 3D models of S-protein ectodomain trimers in two conformational states, a partially ‘open’ (6VYB) and a ‘closed’ (6VXX) state. The AlphaFold2 model was evaluated against each of the three chains (A, B, and C) of both cryo-EM models using the AS2S, US-align, and TM-score servers and the results were reported in Table 2. Alignments of structures are shown in Figure 5, red regions indicate good structural homology, whereas grey regions fall outside of the 5 Å RMSD threshold. For the open-state model, the best and worst scores corresponded to chain A scoring 77.019 GDT-TS and chain B scoring 65.859, respectively (Table S3). The closed-state model produced very consistent scores of ~74.4 GDT-TS, with a difference of less than 0.1 GDT-TS. Aside from chain B in the open state, all chains had scores above 70, which indicates very high structural similarity. AlphaFold2 models performed better with closed-state conformations with one exception (chain A). TM scores were above 0.8 and 0.9 for normalized lengths of the AlphaFold2 and cryo-EM models, respectively, except for 6VYB-Chain B that had a TM score under 0.7 for the normalized length of the AlphaFold2 model. For atoms falling under the 5 Å RMSD threshold, all models had an RMSD of around ~2.1 Å for 800–900 residues, except for 6VYB-Chain B which had an RMSD of 1.43 Å (spanning 711 residues excluding the entirety of the RBD in the up (open) conformation). These scores suggest that the AlphaFold2 structures have highly accurate topologies that can be used confidently for research purposes. They can significantly reduce the wait times for accurate 3D models. To further extend the validation, we used only the individual NTD, RBD, and S2 subunit regions against the high-scoring 6VXX-A and 6VYB-A entries (Figure S3). Overall, AlphaFold2 was able to model the S2 subunit the best (TM scores ~1), followed by the NTD (TM scores of ~0.98), and lastly the RBD (TM scores of ~0.92) (Table S3).

In both the open and closed states, the S2 subunit showed the least number of structural deviations, while the S1 subunit contained the bulk of the structural deviations, with the NTD exhibiting more structural differences than the RBD except for the RBD in its open state (6VYB-B). Walls et al. [16] reported that the S2 subunit had a better resolution than the S1 subunit, which could be explained by the heterogeneity introduced by the two differing conformations at the S1 subunit. The closed state exhibited higher scores (aside from chain A) on every metric, which could prove relevant to the functional role played by the modeled structure. One limitation is the lack of post-transcriptional modifications (PTM) on the modeled structure, but since the observed cleavages occur in a few disordered loop regions, we would expect to see higher alignment scores with PTMs. Gui et al. [37] emphasized the importance of the open conformational states of SARS-CoV for ACE2 receptor binding when compared to the closed state, which imposes restrictions on the strategies that can be used for vaccine and drug targeting.

## 4. Discussion

Intriguing patterns of mutational and structural recruitment across all VOCs and haplotypes suggest convergent evolutionary processes manifesting along the pandemic. In fact, Tomaszewski et al. [38] were the first to study the prevalence of mutations making up VOC constellations in different regions of Australia and the effects that latitude had on the establishment of VOC-induced disease. They tracked massive increases in prevalence of VOC Omicron mutations and haplotypes occurring a few weeks after the first two reported cases in Sydney and Darwin on 28–29 November 2021, only a month following its first appearance in Botswana and South Africa. They showed that the molecular profile of VOC Omicron replaced the fully prevalent VOC Delta in less than a month but that the constellation did not appear monolithically in different regions of the continent. Remarkably, many of its mutations were already present in Australia during the first year of the pandemic. The fulminant emergence of VOC Omicron and haplotypes in a country with one of the highest vaccination rates and toughest restriction policies on the planet, including closed international and regional borders, lockdowns, extensive contact tracing, and strong mandatory quarantine restrictions, strongly favors convergent adaptive scenarios of VOC emergence over evolutionary founder effects or super-spreader events. This conclusion is also supported by a recent study focusing on the younger lineages of VOC Omicron where five mutations affecting the S-protein were inherited across five subvariants in a process that increased immune evasion, especially in the BQ.1.1 lineage of concern [39]. The recruitment of earlier haplotypes into later VOCs and of specific structures of affected regions across haplotypes also supports the piecemeal emergence of VOC constellations, with mutations occurring additively across time. In response to external pressures such as global mitigation efforts, the virus explores mutations and structures that improve fitness in a process of entropy reversals and fixation at different sites of the S-protein. This process has been shown to favor changes that improve S-protein stability and interactions with the hACE2 receptor [40].

Our comparative structural analysis of S-protein structures from VOCs and their corresponding haplotypes uncovered interesting patterns of structural change. The regions involved included numerous loops or regions of intrinsic disorder, many of which hosted epitopes for antibody binding that are of immunological importance. The changes introduced to these specific regions also showed patterns of recruitment across the timeline of the pandemic, showing how different regional conformations impact viral survivability and infectiousness. These regional changes showed high frequency at the NTD supersite, and such haplotypes had a higher impact on seasonal behavior. Lastly, we describe patterns of recruitment observed for the three major VOCs, with VOCs Alpha and Delta behaving synergistically and VOC Omicron behaving antagonistically with their corresponding haplotypes.

### 4.1. Structural Changes Involve Regions of Protein Flexibility

Structure determines function for the vast majority of proteins [41]. Consequently, evolutionary changes at the protein structural level often indicate necessary adaptations for better molecular performance. Remarkably, many of the regions of high structural change we identified occur in loops that are considered disordered regions, such as the N2 and N4 loops of NTD (regions 2 and 6, respectively) and the S1/S2 boundary of region 16. Regions 2, 5, 6, 16, and 19 (N2, N3, N4, S1/S2 boundary, residues 829–851), along with multiple regions in the RBD, were designated as regions of high flexibility given their high disordered propensity scores [27,42,43]. Many of these disordered regions play vital roles in protein function or are structurally relevant, such as regions 2, 5, and 6 being critical to the NTD supersite and the S1/S2 boundary that harbors the S1/S2 cleavage site being essential for viral infectivity [26,44]. The different combinations of structural change at one region along with changes at multiple regions could help explain factors such as VOC incidence and case fatality rates.

### 4.2. Structural Changes Are Important for Disease Mitigation

The NTD and RBD of the S1 subunit constitute the main targets for vaccine and drug development efforts due to their accessibility, i.e., their ability to engage in interactions with other molecules. The two domains face away from the viral membrane, thus allowing for multiple approach angles and exposure to numerous epitopes that act as neutralizing targets for antibodies [45]. The most critical structures of the NTD involve loops N3 and N5. They define a conformational epitope responsible for viral immune escape [46]. Regions 2, 5, and 6 of the NTD region correspond to loops N2, N3, and N4, as originally defined by Chi et al. (2020) [47]. N2 and N3 harbor deletions RDR1 (Δ69–70) and RDR2 (Δ141–144 and Δ146), respectively. The N3-linked RDR2 completely abolishes 4A8 antibody binding at its epitope [48]. Regions 2 and 5 also include half of the residues involved in the NTD supersite, N74 and N149, respectively, the other two being N17 and N122 (part of observed region 4) [49]. We see a significant structural change in VOCs and haplotypes associated with the N3 loop. In sharp contrast, our study does not reveal significant structural change associated with the N5 loop. Note, however, that region 7 occurs directly after RDR3 (Δ210), which is a deletion located in an area between loops N4 and N5. This region experienced a change in VOC Omicron but failed to show an impact on antibody binding to its respective epitope.

The supersite and structures of the NTD, especially its loops, are very plastic, with the bulk of the galectin fold structure being shaped by the N3 loop [49]. The fact that the NTD sites and structures had some of the lowest observed TM scores in our study (e.g., for VOCs Delta and Omicron, and H8) (Figure 4) supports this plasticity. Note that antibodies targeting the NTD have high neutralizing potential and that the NTD has only a single site of vulnerability. These two properties make the NTD a great target for vaccine and drug development efforts. The only limitation is the restricted approach angle required for antibody binding due to its close proximity to the viral membrane, which limits the surface area available for binding [49].

It has been shown that differences in the NTD loops can impact RBD exposure prior to S1 and S2 separation, thus resulting in differential binding of the S-protein to the hACE2 receptor [50]. The NTD also plays a role in S2′ cleavage and proteolytic activation driving membrane fusion between the S-protein and the host cells [50]. This proteolytic activation is driven by the TMPRSS2 enzyme at the S2′ site after S1/S2 cleavage [51]. This process is highly affected by the NTD region. A recent study compared TMPRSS2 sensitivity between the VOC Kappa variant against a VOC Kappa chimera with a VOC Delta NTD, which showed that the chimeric construct had higher TMPRSS2 sensitivity on par with the VOC Delta variant [52]. However, the same was not true for VOC Omicron. The same chimeric strategy applied to VOC Omicron with the VOC Delta NTD showed a moderate increase, indicating that VOC Omicron is TMPRSS2-independent [53].

Unlike the NTD, the RBD offers multiple approach angles and multiple sites of vulnerability [49]. This provides more options when it comes to neutralizing strategies and techniques. We found that the majority of the structural deviations in the RBD were translations in 3D space. However, a subregion of the N1 loop, residues 364–372, was the only segment capable of twisting away from its original axis. This tiny region (a short alpha helix) is part of an RBD cavity that locks the S-protein into a closed pre-fusion state by pushing the F100D and W100E residues of the long CDRH3 arms of C144 into the RBD cavity, thus preventing binding to the ACE2 protein [24]. This locking requires adjacent RBDs to be in a ‘down’ conformation. Residues 364–372 contain the entirety of the second alpha helix and other portions of the cavity, including residues F374 and W436 of the first alpha helix (residues 337–344) [24]. Details of the structural alignments can be examined in Figure S1. In Figure 1, residues 364–372 can be visualized as red bars for haplotypes H5, H4, H7, H8, and H19, all of which adopt the same conformation that twists away from its axis. 

Another S1 segment that experienced notable change was made up of regions 13 and 14, i.e., residues 591–594 and 625–642, respectively. These regions were linked to one of two linear epitope hot regions, specifically S1-88 to S1-114, which ranges from residues 525 to 685 [54]. Region 13 itself does not consist of an epitope but comes into close proximity to epitope S1-97 (residues 577–588) [54]. Region 14 coincides exactly with epitope S1-106/106 (residues 625–642) [54]. A number of different structural variations were present in this region (see Figure S1), which is one of the few regions that showed more than two associated groups in Table 1. Additionally, region 12 (residues 551–575), which consists of the S1-93 epitope (residues 553–564) [54], was found mainly altered in VOC Omicron (Figure 2), even though all variants show some level of 3D transformation that fails to cross the RMSD threshold of Figure 1. Region 12 also hosts the S14P5 epitope (residues 553–571), which is relatively well conserved and has neutralizing capabilities as it causes steric hindrance close to the RBD, limiting binding to the ACE2 receptor [55,56,57]. Thus, protein structural changes seem to be influenced by IgG antibody activity, probably because antibody binding is an environmental pressure working against the virus which pushes the virus to evolve at contact points to improve survival.

While the NTD and RBD remain the main targets for vaccine and drug development [45], we know, however, that antibodies exist that bind to the S2 subunit as well, which is responsible for virus–host membrane fusion [47]. Fusion is initiated by inserting the Fusion Peptide (FP) into the host membrane [58], assisted by the structural rearrangement of the S2 subunit after the cleavage of the S1 subunit [45]. This structural rearrangement is driven by the binding of HR1 and HR2 regions to form a 6-α-helix bundle [59]. Surprisingly, very few changes were observed in the S2 subunit showing RMSD or TM scores of significance. Only regions 18 (within the FP) and 19 were selected for analysis as they exhibited clear deviations (see numerous red bars in Figure 1). The S2 subunit thus appears relatively well conserved when compared to the S1 subunit. The other linear epitope hot region mentioned in the study by Li et al. [54] spanned residues 770–829, including the entirety of region 18 (FP) and the very first residue of region 19. Region 18 was mostly impacted by haplotypes H3 and H12 along with the VOC Omicron constellation (Figure S1). Again, we see that many of these structural changes tend to coincide with epitopes. Regions 18 and 19 likely influence epitope S21P2 (residues 808–827), which was one of the two linear epitopes explored in the earlier stages of the pandemic along with S14P5 [55,56]. S21P2 is in close proximity to the FP site that is responsible for membrane fusion. Antibody binding at this site can hinder the fusion process and thus neutralize the virus particle. Such behavior was observed when targeting the fusion regions of SARS-CoV-1 and MERS-CoV [60]. While S14P5 and S21P2 are known to be well conserved [56,61], S14P5 experienced structural changes in VOC Omicron (Figure 1). In contrast, S21P2 retained its conserved status by exhibiting only minor changes evident at the 1–2 Å RMSD range. Thus, S21P2 appeared structurally very stable during the pandemic. The neutralizing capabilities of this region supports its significance in efforts of vaccine development.

Finally, the S1/S2 boundary (region 16) also experienced notable change identified by very low RMSD and TM scores. This region was impacted by mutations such as P681H in VOCs Alpha and Omicron or P681R in VOC Delta. However, only VOC Omicron seems to exhibit high structural change, which is likely due to the N679K mutation that is part of haplotype H12. This region is important for infectivity as it contains an RRAR motif (residues 684–686). This motif, when deleted, causes the virus to replicate slower in human lung epithelium cells as it now undergoes endosomal fusion rather than the plasma membrane fusion pathway, which is more efficient [44]. The RRAR motif appears right before the predicted cleavage site of the S1/S2 subunits, which is acted upon by the furin protease enzyme [26].

This Furin cleavage site (FCS) is a unique feature of viral transmission [44,62]) and pathogenicity [63] that differentiates SARS-CoV-2 from other sarbecoviruses [13]. Changes to the S1/S2 boundary and the FCS were indeed linked to improved viral spread for all variants. However, they were not the primary factor for increased transmissibility of VOC Omicron, as the variant utilized alternative entry mechanisms [13,53]. Our structural analysis of the region reveals that VOCs Alpha and Delta do not differ greatly from the Wuhan model, whereas VOC Omicron shows drastic shifts. These structural changes could explain the difference in transmission rates observed for VOC Omicron against prior variants. They may also be the reason why VOC Omicron appears to be more poorly cleaved than VOC Delta and the Wuhan strain [53]. The N679K mutation, which was found in all VOC Omicron sequences, appears linked to reduced spike expression levels in host cells and showed a preference for transmission through the upper airways when compared to the Wuhan strain [64]. However, the other mutations in VOC Omicron such as N655Y and P681H have been shown to increase spike processing. Consequently, the interaction of these mutations along with their structural impact poses a combinatorial effect, which in turn aids VOC Omicron transmissibility while reducing pathogenicity [64]. From an evolutionary viewpoint, this effect appears crucial for viral persistence, as virus prevalence can only be sustained if the virus can spread effectively without killing the host.

### 4.3. Recruitments of Structural Changes That Improve Molecular Function Appear Pervasive

The space of structural recruitment events across the S-protein throughout the pandemic can be thought of as a combinatorial problem where each VOC and haplotype adopts specific sub-structures in various combinations. Adoptions of earlier structures in later VOCs and haplotypes reveal not only important regions but highlight the importance of conformation reversals in the evolution of viruses that indicate prior structures were more fit for viral survival. The space of 3D exploration, or rather structural entropy, is apparent with the examples of some regions of the S-protein in haplotypes H5, H4, H7, H8, and H19 adopting similar conformations while still retaining uniquely characterizing conformations of other protein regions (Table 1). The concept of mutational entropy and the expansion or reversal of entropy at a specific amino acid position along the S-protein sequence [27] can be extended to protein structure by identifying continuous change or stability of structural conformations at specific regions in the form of a structural entropy. Each region identified harbors its own entropic patterns, with changes in structure being considered entropy expansion and recruitment of past structures being considered entropy reversals.

### 4.4. Haplotypes Altering NTD Structure Impact Seasonal Behavior

The NTD was identified as a region of interest in our study because of the constant flux of structural conformations involving the N loops of its supersite, which are targeted by highly protective and specific antibodies [49,65,66]. Figure 1 and Figure 3b clearly show the extent to which the NTD region experienced targeted structural change. This fact is particularly important because a study of the impact of latitude and hemispheres on the rise of mutant constellations has linked the NTD to SARS-CoV-2 seasonal behavior [25,27]. Note that worldwide correlations and time-series correlation charts provide strong support for the seasonal behavior of COVID-19 throughout the pandemic [67,68]. This should not be considered surprising. SARS-CoV-2 belongs to a family of coronaviruses that are seasonal in nature, with peak incidence rates coinciding with the winter months (peaking in January to March) and lower transmission rates unfolding during the summer [69,70]. This pattern of seasonal behavior, however, is much less conserved for tropical climates, revealing a complex interplay between climate and latitude [71,72,73].

Tomaszewski et al. [14] explored mutant prevalence levels of latitude-delimited haplotypes with mutation accumulation plots unfolding along the pandemic. This exploration allows us to find links between haplotypes that alter the structure of the NTD and prevalence patterns that are indicative of seasonal behavior. Haplotype H3 makes a good case for the seasonal impact of the NTD region. Compared to other haplotypes, H3 significantly alters the structure of the N loops while not affecting the structure of other binding domain regions. Prevalence plots of H3 across different climactic zones uncovered seasonal and latitudinal links [14]. Plots were seen to go up and down according to the seasons, with winter seeing the highest rates and the tropical regions seeing much lower prevalence levels than Arctic and Northern temperate regions. Similarly, the difference in prevalence between haplotypes H8 and H7 also showed seasonal links. Our structural analysis revealed that H8 alters the structure of both the NTD and RBD while H7 only affects the RBD. Remarkably, a notable difference in prevalence levels exists between the haplotypes for tropical regions whereas H8 showed lower prevalence levels than H7 [14].

Clearly, haplotypes that impact the NTD structure are more likely to be involved in seasonal behavior than those that do not. The NTD structure, which in our study was the site of the majority of structural change, harbors a galectin-like fold. One of the major functions of galectins is immune response regulation [74,75]. Interestingly, galectin-like proteins (lectins) involved in preventing bleaching in scleractinian corals were found to function best in an optimal temperature range of 25–30 °C, beyond which their binding capability decreased rapidly, blocking the binding and recognition of pathogens and leading to coral vulnerability [76,77]. This temperature-dependent behavior of galectin-like proteins could extend to SARS-COV-2, with the NTD and RBD both behaving differently. The NTD sees continued flexibility at higher temperatures while the RBD adopts a closed conformation that blocks ACE2 binding and affects haplotype prevalence in the population [14,78]. This suggests that while the RBD remains closed, the NTD could still play a role in host immune evasion responses, as the NTD is shown to be quite similar to human receptors such as Ephrins, suggestive of the NTD’s versatile binding to other human proteins [78].

### 4.5. Patterns of Molecular Evolution Can Be Synergistic or Antagonistic

The analysis of haplotypes unfolded a history that revealed predictive patterns of structural change for future VOCs and haplotypes (Figure 1). We find, for example, that haplotype H3 was a good indicator for structural changes to come with the rise of VOC Alpha. The combination of haplotypes had particularly good predictive value. Even smaller structural changes that did not cross the RMSD threshold later turned into regions of significant structural change, as seen with the small regions spanning the N2 and N3 loops of NTD in haplotypes H5, H4, H3, H7, H8, and H19. These regions later passed the 3.0 Å threshold of structural change in VOC Omicron. The numerous changes in the RBD would also have been neglected if we only explored VOC definitions in AlphaFold2-simulated structures. Both VOCs Alpha and Delta, which showed little to no deviation in the RBD region, harbored haplotypes that did exhibit significant structural change, spanning in part almost the entirety of the RBD. This behavior was a later predictor for VOC Omicron, as the entire RBD region experienced a translation in 3D space without much change to the inherent structures (Figure S1).

We dissected these haplotype recruitment behaviors into two categories: synergistic and antagonistic. VOCs Alpha and Delta behaved antagonistically by suppressing the individual effects of their constituent haplotypes. In other words, VOCs Alpha and Delta experienced fewer regional changes than their corresponding haplotypes. While VOC Alpha only showed changes altering the N2 and N3 loops of the NTD, its corresponding haplotypes H3, H4, and H5 also impacted the S1/S2 boundary and region 19, H3 and H4 altered the N4 loop in the NTD, and H4 and H5 introduced numerous changes to the RBD. VOC Delta behaved very similarly. While structural changes were confined to the N3 loop of the NTD, all its constituent haplotypes (H8, H7, and H5) introduced changes in the RBD region, and H8 also impacted the N4 loop. This antagonistic behavior appears to be acting as a buffer to counteract the effects of structural recruitments that fail to provide a greater benefit to viral success. In sharp contrast, VOC Omicron stood out when it came to recruitment patterns as it amplified and collected the individual changes introduced by its constituent haplotypes. Haplotypes H19, H15, H3, H4, and H5 introduced changes that contributed to the regions of change observed in VOC Omicron. While VOCs Alpha and Delta counteracted the effects of their individual haplotypes (even if numerous haplotypes were introducing the same change), VOC Omicron incorporated even the slightest haplotypic structural change into its makeup. For example, the N3 loop of the NTD was only impacted by one of VOC Omicron’s constituent haplotypes (H3) but was still kept (though not the exact same change as H3). VOC Omicron synergistically amplified the impact of its haplotypes to change the entirety of the NTD supersite and the RBD. The only exception was region 19, which saw a slightly lower change in VOC Omicron than its corresponding haplotypes. This reveals an added layer of complexity which could be due to the vastly larger number of mutations forming the VOC Omicron constellation.

The reason why earlier haplotypes behaved antagonistically to suppress the effects of their constituent haplotypes at select regions and why VOC Omicron behaved synergistically to either amplify or collect the effect of its constituent haplotypes remains mysterious. This issue is likely a problem of combinatorics and evolutionary pressures that push the virus to adopt conformations that can improve survival rates within the host population. Possibly, the numerous initial structural conformations did not provide as much of an evolutionary benefit to virus survival. Once enough structural changes accumulated in the emerging VOC Omicron, the entire collection of structural conformations became synergistically more beneficial to the virus, thus enabling the massive recruitment of mutations that gave rise to VOC Omicron. This contrasts with the antagonistic effects observed in the haplotypes of prior VOCs.

## 5. Conclusions

SARS-CoV2 haplotypes reflect seasonal behavior and provide insight into possible biological and evolutionary drivers of protein structural change [14]. Here, we report that functionally important regions along the length of the S-protein exhibited significant levels of structural change when folding was modeled with AlphaFold2. Remarkably, our analysis uncovered patterns of stability interleaved with periods of structural exploration. Not only did haplotype definitions uncover molecular evolutionary patterns, but earlier haplotypes constituted most of the structural changes later observed. These patterns of structural change were repeatedly observed throughout different periods of the pandemic, revealing the importance of the recruitment of those regions to viral persistence, especially the N2-N4 loops of the NTD, the RBD and its translation movement, and the changes observed at the S1/S2 boundary. We found that predictions of future S-protein structures are possible as many changes experienced by earlier haplotypes were also observed in later VOCs and haplotypes. Since these regions experienced significant change, it can be inferred that they experienced significant evolutionary pressures. This makes these regions ideal targets for vaccine and drug development efforts and effective scaffolds for countering disease progression. 

Our study described how haplotypes linked to seasonal behavior impacted S-protein structure. When studying VOCs, it becomes impossible to pinpoint which regions cause seasonal behavior as they represent the culmination of their mutant constellations. However, with haplotypes, we have smaller sets of interacting mutant sites that allow us to better understand how different structural changes contribute to SARS-CoV2 seasonal behavior. This revealed that haplotypes that impacted the structure of the NTD followed the epidemic calendar of the virus, while other haplotypes either did not or followed trends that were not as strong. 

An interesting revelation of our analyses was the difference in recruitment strategies exhibited by major VOCs. VOCs Alpha and Delta behaved antagonistically to suppress many of the changes brought about by their constituent haplotypes. In sharp contrast, VOC Omicron synergistically amplified haplotype effects by recruiting prior structures or introducing new changes. The countless differences observed in our AlphaFold2-enabled haplotypic analysis allude to its importance for future virus studies, especially when cryo-EM structures of proteins are not available. VOC definitions do not encompass the reality of the viral disease landscape and an immense amount of information is generally lost that could help identify regions of functional significance that are subjected to the highest levels of evolutionary pressure. Future work would be needed to expand this analysis to the entire SARS-CoV2 protein repertoire. A molecular dynamic (MD) simulation approach could also unravel the impact of these structural changes on protein dynamics and crucial protein–protein interactions.

## Figures and Tables

**Table 1:** Recruitment of structures ^1^.

V/H	2	5	6	7	9	10	11	13	14	16	18	19
Omicron	1.75	6	3	2	2	2	2	2	4	4	2	3
H16	0.5	0.25	0	0	0	0	0	0	0	0	0	0
H17	0	0	0	1	0	0	0	0	0	0.5	0	0
H14	0	5	0	0	0	0	0	0	0	0	0	0
H19	0.5	0	1	0	1	1	1	1	1	1	0	1
H13	0	0	0	0	0	0	0	0	0	0	0	0
H15	2	0	0	0	0	0	0	0	3	0	0	0
H12	0	0	0	0	0	0	0	0	0	3	0.5	0
Delta	0	4	0	0	0	0	0	0	0	0.5	0	0
H8	0.5	3	1	0	1.5	1.5	1.5	1	1	1	0	1
H7	0.5	0	0	0	1	1	1	1	1	1	0	1
Alpha	1	2	0	0	0	0	0	0	0	0.5	0	0
H1	0	0	0	0	0	0	0	0	0	0.5	0	0
H3	1.5	1	2	0	0	0	0	0	2	2	1	2
H4	0.5	0	1	0	1	1	1	1	1	1	0	1
H5	0.5	0	0	0	1	1	1	1	1	1	0	1

^1^ Rows describe VOCs (V) and haplotypes (H) arranged in chronological order following their time of emergence during the pandemic. Columns describe structural regions along the S-protein. Numbers describe levels of deviation from the Wuhan structures.

**Table 2:** Benchmarking results.

State-Chain	LGDT-TS (AS2S)	TM Score/L (US-Align Server)	Superimposed RMSD < 5 Å (TM-Score Server)
Closed-A (6VXX-A) L = 972	74.434	*L1: 0.82683, L2: 0.94899	2.10/884
Closed-B (6VXX-B) L = 972	74.357	0.82682, 0.94899	2.10/884
Closed-C (6VXX-C) L = 972	74.357	0.82682, 0.94899	2.10/884
Open-A (6VYB-A) L = 966	77.019	0.82432, 0.95200	2.07/894
Open-B (6VYB-B) L = 944	65.859	0.69501, 0.81421	1.43/711
Open-C (6VYB-C) L = 960	71.146	0.80577, 0.93502	2.26/836

*L1 represents the length of the AlphaFold2 reference (Wuhan WT strain) model (1121 amino acids) superimposed onto all other cryo-EM structures.

## Data Availability

The data presented in this study are openly available in ModelArchive under accession *ma-gca-sprot* (https://www.modelarchive.org/doi/10.5452/ma-gca-sprot; Released on 15 January 2024). Other data and information supporting the findings of this study are either public or available within the article and its Supplementary Materials.

## Supplementary Materials

The following supporting information can be downloaded at: https://www.mdpi.com/article/10.3390/biology13030134/s1. Figure S1: Structural models of 12 regions (regions 2, 5–7, 9–11, 14, 16, 18, and 19) of high structural change showcasing the atomic level differences for each VOC and haplotype. Distances between corresponding amino acids of the Wuhan reference against the VOC or haplotype are represented through dashed lines and measurements are in Ångstroms. Figure S2: Structural models of S-proteins from VOC Alpha (a) and VOC Delta (b) with regions of structural change mapped onto them and colored according to TM scores and Chimera alignments. Figure S3: Structural alignments of AlphaFold2-modeled NTD, RBD, and S2 domains against cryo-EM structures 6VXX-A (a) and 6VYB-A (b). Table S1: Emergence of VOCs and haplotypes and their mutant constellations. Time of origin is given in a scale of relative appearance. Data from ref. [14]. Table S2: Pruned and total aligned residues and RMSD values for structural alignments of each lower-ranked model against rank 1 structures and all ranked models against the Wuhan modelled structure. Table S3: Benchmarking results for three domain regions of the modeled Wuhan strain S-protein structure against each of three experimentally determined S-protein structures, which were identified using PDB accession codes.
