# Supplementary material for: AlphaFold2 Reveals Structural Patterns of Seasonal Haplotype Diversification in SARS-CoV-2 Spike Protein Variants
Source: Biology (Basel). 2024 Feb 21;13(3):134. doi: 10.3390/biology13030134 (PMC10968544; doi:10.3390/biology13030134)
Supplement: Supplementary file 1 [file biology-13-00134-s001.zip › biology-2841665-supplementary/Supplementary/Figure S1.pdf]

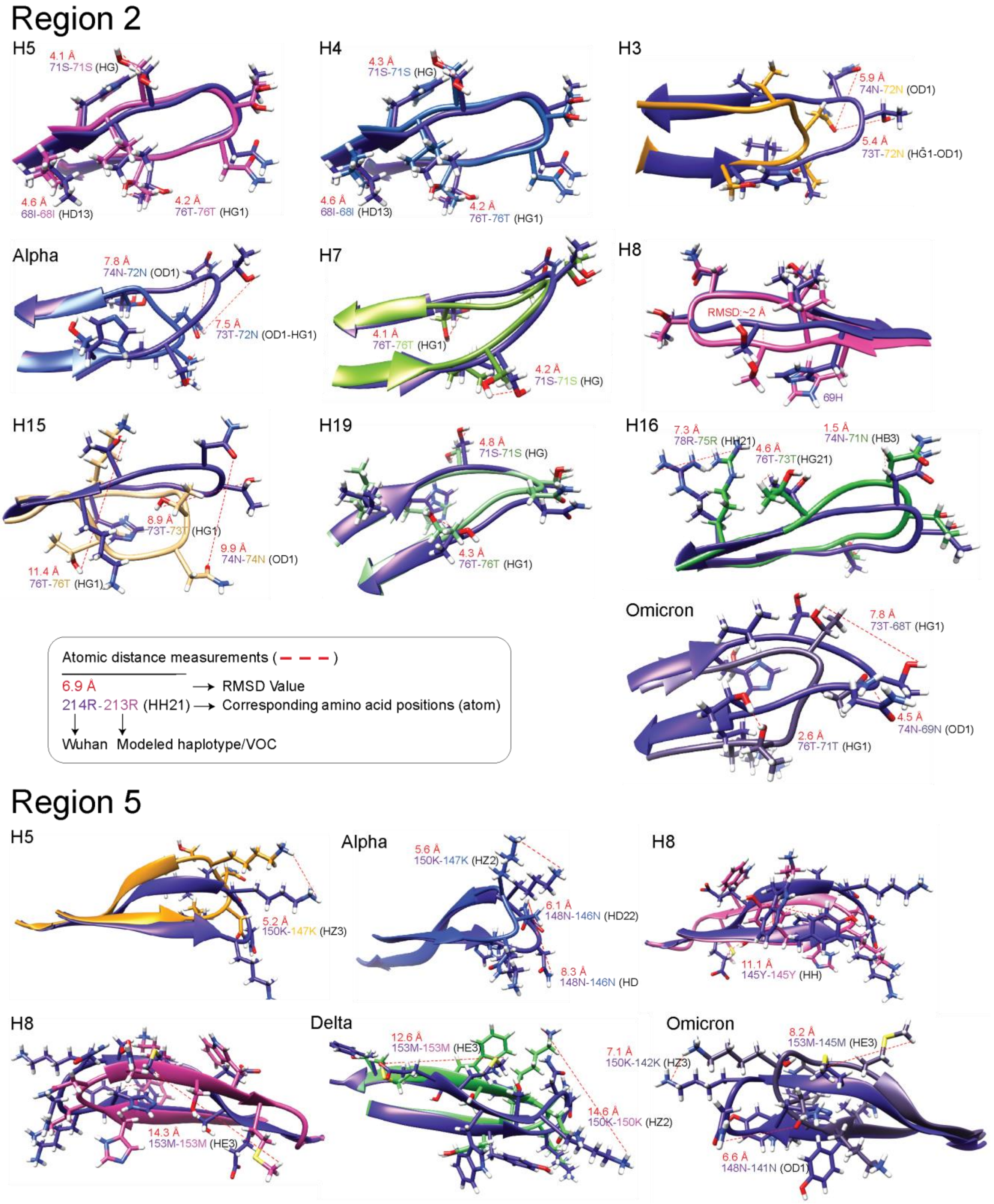

Figure S1. Structural models of 12 regions (region 2, 5-7, 9-11, 14, 16, 18, and 19) of high structural change showcasing the atomic level differences for each VOC and haplotype. Distances between corresponding amino acids of the Wuhan reference against the VOC or haplotype are represented through dashed lines and measurements are in Ångstrom.

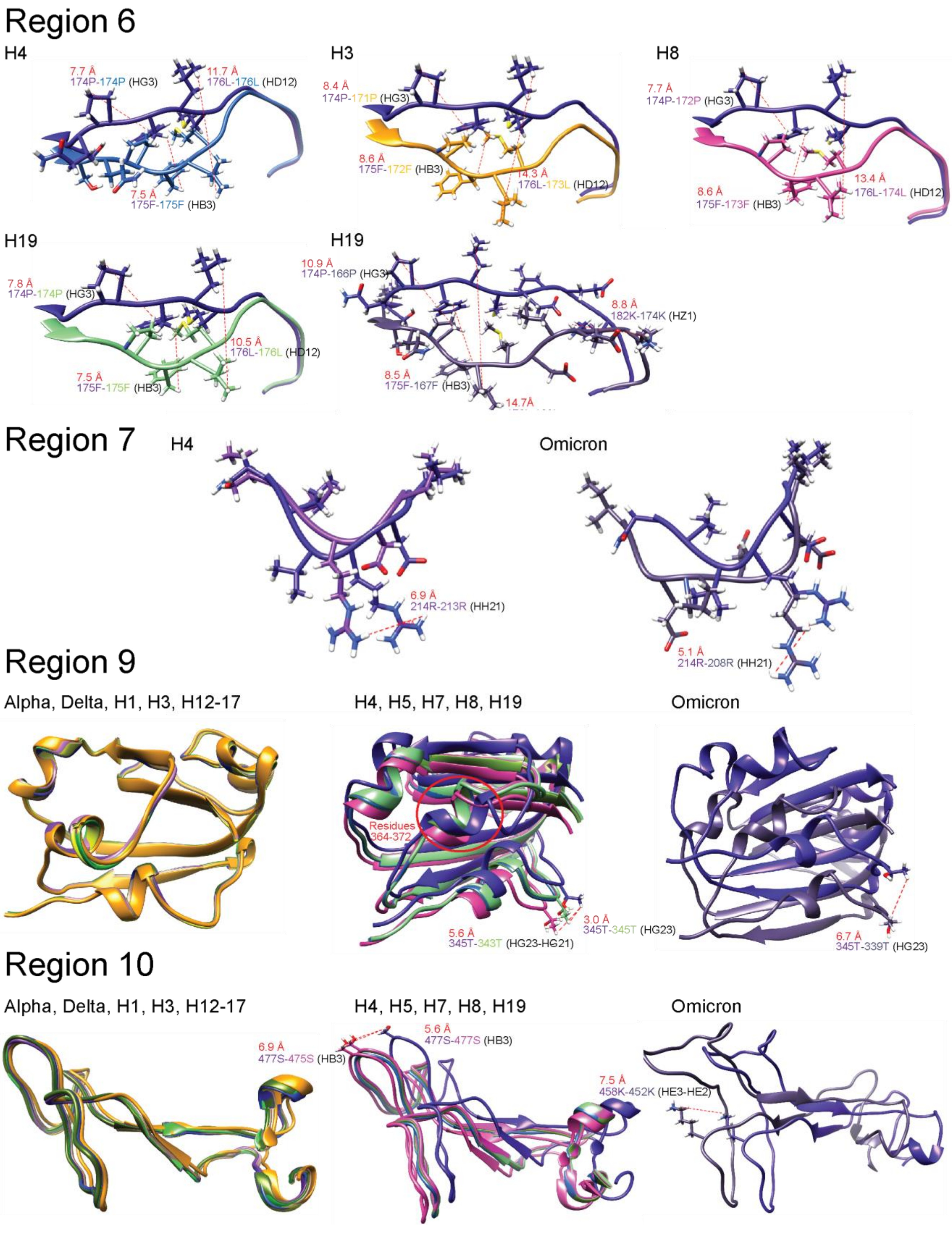

Figure S1. (continued)

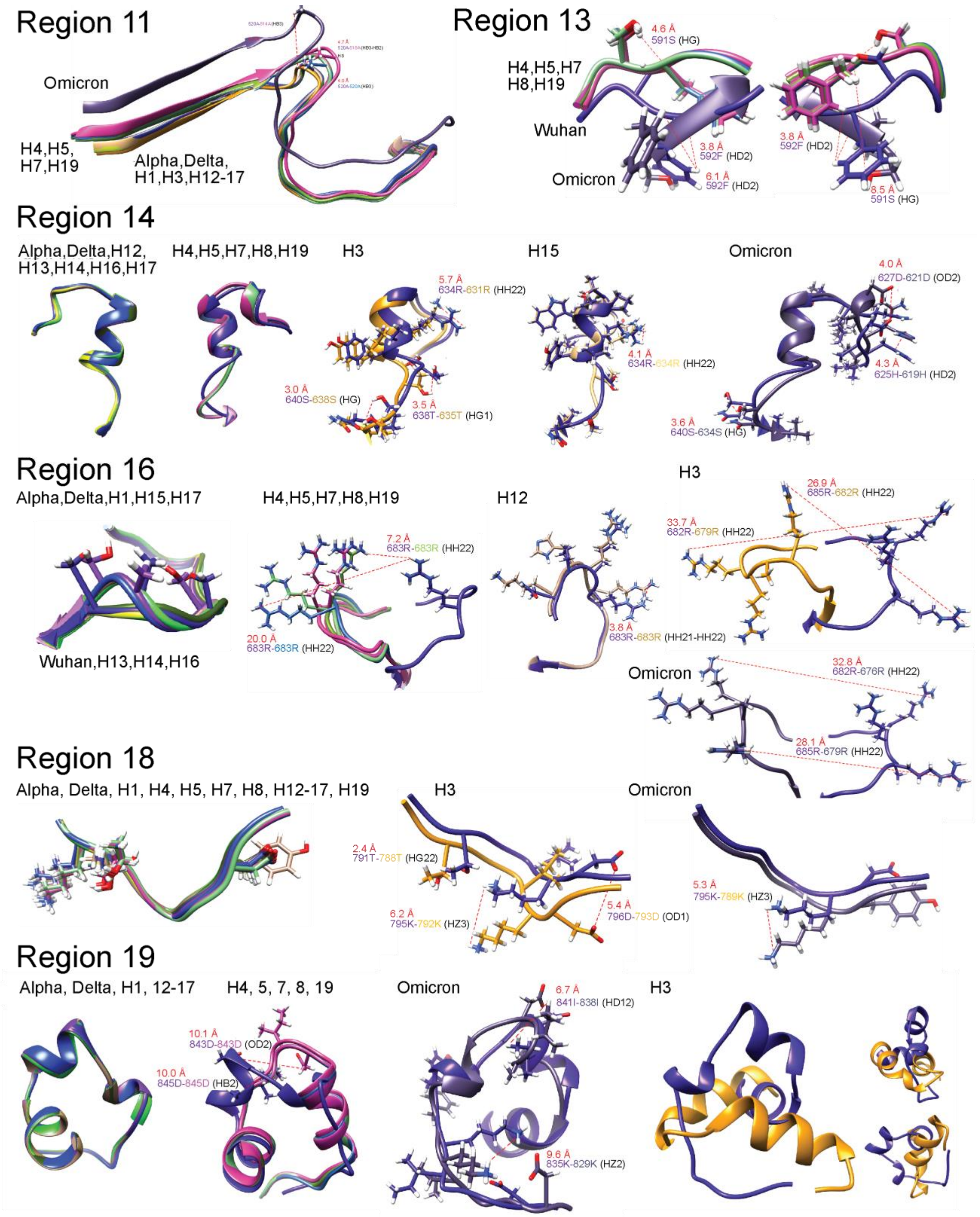

Figure S1. (continued)
