# Supplementary material for: AlphaFold2 Reveals Structural Patterns of Seasonal Haplotype Diversification in SARS-CoV-2 Spike Protein Variants
Source: Biology (Basel). 2024 Feb 21;13(3):134. doi: 10.3390/biology13030134 (PMC10968544; doi:10.3390/biology13030134)
Supplement: Supplementary file 1 [file biology-13-00134-s001.zip › biology-2841665-supplementary/Supplementary/Figure S2.pdf]

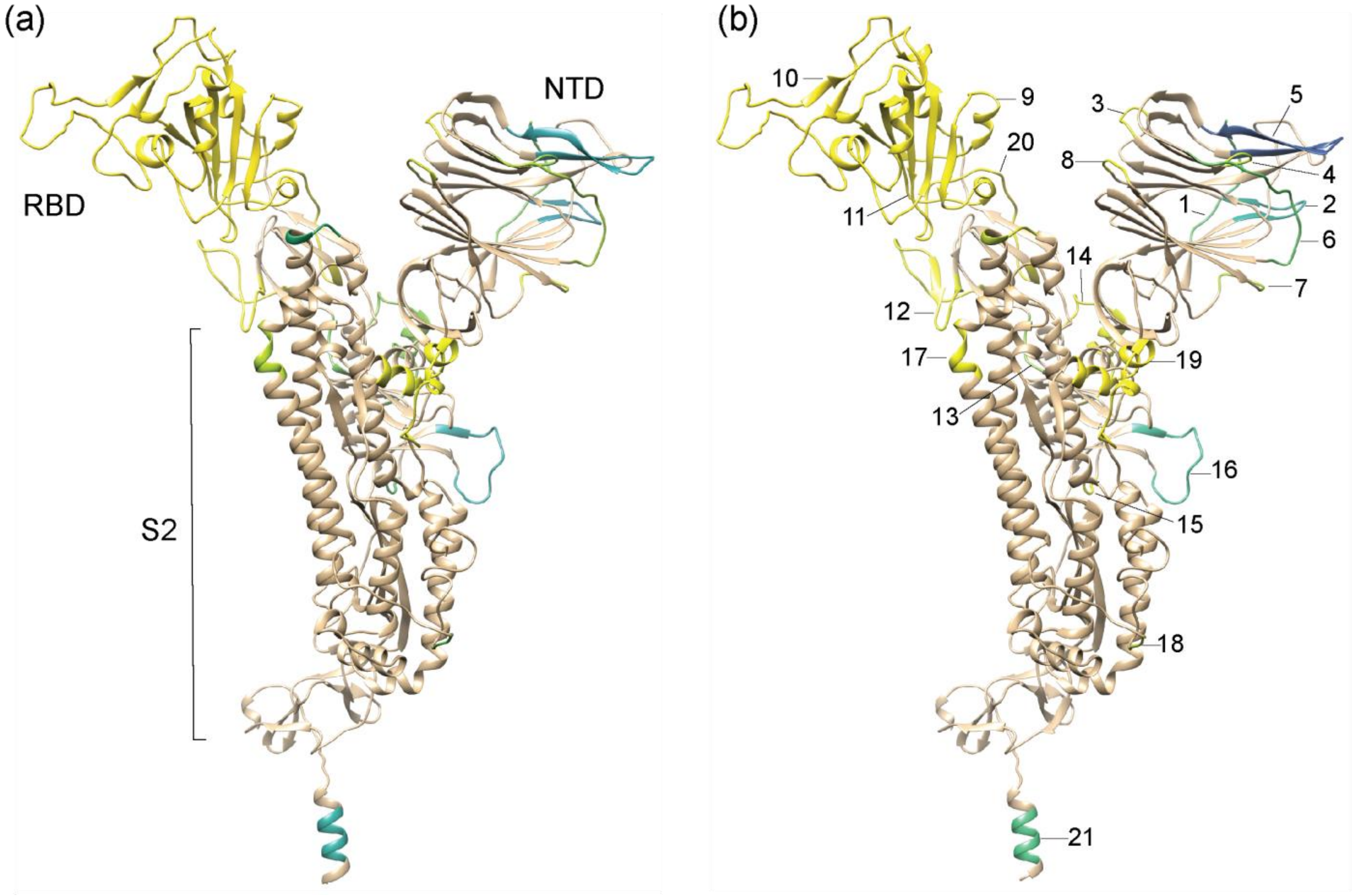

Figure S2. Structural models of S-proteins from VOC Alpha **(a)** and VOC Delta **(b)** with regions of structural change mapped onto them and colored according to TM scores and Chimera alignments. Numbers indicate regions of structural change.
