# Supplementary material for: AlphaFold2 Reveals Structural Patterns of Seasonal Haplotype Diversification in SARS-CoV-2 Spike Protein Variants
Source: Biology (Basel). 2024 Feb 21;13(3):134. doi: 10.3390/biology13030134 (PMC10968544; doi:10.3390/biology13030134)
Supplement: Supplementary file 1 [file biology-13-00134-s001.zip › biology-2841665-supplementary/Supplementary/Figure S3.pdf]

biology

MDPI

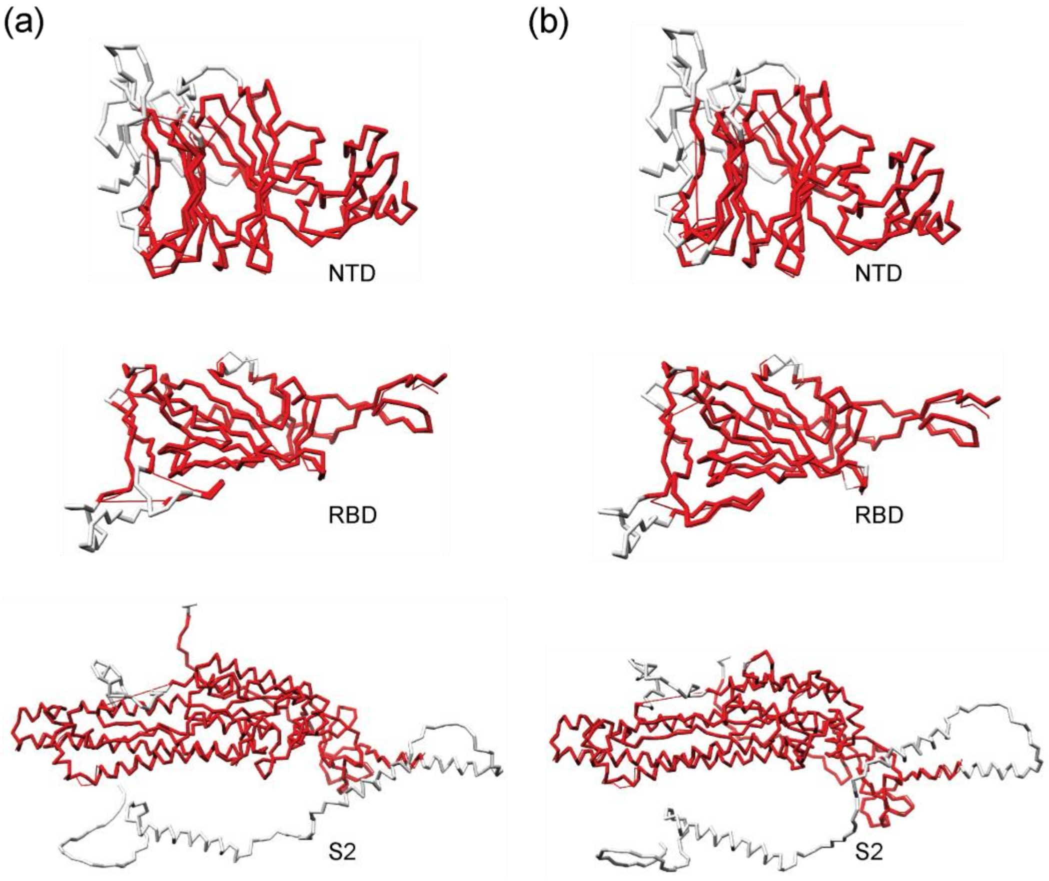

Figure S3. Structural alignments of AlphaFold2 structures against cryo-EM structures 6VXX-A **(a)** and 6VYB-A **(b)**.
