# Supplementary material for: AlphaFold2 Reveals Structural Patterns of Seasonal Haplotype Diversification in SARS-CoV-2 Spike Protein Variants
Source: Biology (Basel). 2024 Feb 21;13(3):134. doi: 10.3390/biology13030134 (PMC10968544; doi:10.3390/biology13030134)
Supplement: Supplementary file 1 [file biology-13-00134-s001.zip › biology-2841665-supplementary/Supplementary/Table S1.pdf]

biology MDPI

Table S1. Emergence of VOCs and haplotypes and their mutant constellations. Time of origin is given in a scale of relative appearance. Data from ref. [14].

Haplotype/VOC	Mutations	Time of origin
**Omicron**	T19I, L24-, P25-, P26-, A27S, A67V, H69-, V70-, T95I, G142D, V143-, Y144-, Y145-, N211-, L212I, V213G, +214EPE, G339D, R346K, S371F, S373P, S375F, T376A, D405N, R408S, K417N, N440K, G446S, L452R, S477N, T478K, E484A, Q493R, Q493R, G496S, N501Y, Y505H, T547K, D614G, H655Y, N679K, P681H, N764K, D796Y, N856K, Q954H, N969K, L981F	16
**H16**	L24-, P25-, P26-, A27S, V213G, S371F, T376A, D405N, R408S	15
**H17**	N211-, L212I, S371L	14
**H14**	A67V, V143-, Y145-, G496S, T547K, N856K, L981F	13
**H19**	S373P, S375F, K417N, S477N	12
**H13**	E484A, Q493R, Y505H	11
**H15**	N764K	10
**H12**	G339D, H655Y, N679K, P681H, D796Y, Q954H, N969K	9
**Delta**	T19R, T95I, G142D, E156G, F157-, R158-, A222V, L452R, T478K, D614G, P681R, D950N	8
**H8**	E156G, R158-, F157-	7
**H7**	T19R, P681R	6
**Alpha**	H69-, V70-, Y144-, N501Y, A570D, D614G, P681H, T716I, S982A, D1118H	5
**H1**	A570D, T716I, S982A, D1118H	4
**H3**	H69-, V70-, Y144-	3
**H4**	N501Y	2
**H5**	D614G	1
**Wuhan**	–	0
