# Supplementary material for: AlphaFold2 Reveals Structural Patterns of Seasonal Haplotype Diversification in SARS-CoV-2 Spike Protein Variants
Source: Biology (Basel). 2024 Feb 21;13(3):134. doi: 10.3390/biology13030134 (PMC10968544; doi:10.3390/biology13030134)
Supplement: Supplementary file 1 [file biology-13-00134-s001.zip › biology-2841665-supplementary/Supplementary/Table S2.pdf]

Table S2. Pruned and total aligned residues and RMSD values for structural alignments of each lower-ranked model against rank 1 structures and all ranked models against the Wuhan modelled structure.

VOC/Haplotype	Rank #	Rank #1				Wuhan			
		Pruned	RMSD	Total	RMSD	Pruned	RMSD	Total	RMSD
**OmicronS371F**	1					634	1.15	1265	12.494
	2	1112	0.552	1267	12.215	647	1.175	1265	7.973
	3	219	0.898	1267	39.107	176	0.978	1265	36.523
	4	280	1.051	1267	46.479	291	1.088	1265	43.485
	5	210	0.744	1267	37.782	219	0.976	1265	41.519
**H16**	1					1141	0.2	1270	2.282
	2	1137	0.375	1270	8.337	1126	0.387	1270	8.309
	3	326	0.981	1270	40.32	324	1.027	1270	40.868
	4	261	0.714	1270	41.939	250	0.715	1270	41.959
	5	143	0.661	1270	40.384	137	0.639	1270	40.455
**H17**	1					1138	0.181	1272	5.573
	2	1131	0.415	1272	10.05	1134	0.399	1272	8.446
	3	301	0.952	1272	43.14	296	0.971	1272	42.055
	4	263	1.099	1272	42.012	233	1.051	1272	41.127
	5	276	0.894	1272	37.412	259	0.875	1272	35.86
**H14**	1					1144	0.219	1271	2.665
	2	1139	0.434	1271	8.885	1138	0.461	1271	8.684
	3	333	1.054	1271	39.332	333	1.076	1271	39.059
	4	262	0.89	1271	41.731	248	0.776	1271	41.344
	5	260	0.764	1271	33.83	256	0.749	1271	33.686
**H19**	1					930	0.78	1273	8.936
	2	1127	0.706	1273	13.063	869	0.8	1273	10.323
	3	306	0.941	1273	42.768	276	1.022	1273	40.774
	4	226	0.953	1273	43.279	208	0.925	1273	40.015
	5	306	0.998	1273	39.389	309	1.114	1273	37.685
**H13**	1					1217	0.379	1273	1.274
	2	1140	0.423	1273	8.946	1138	0.436	1273	8.937
	3	330	0.996	1273	39.296	325	0.99	1273	39.447
	4	259	0.932	1273	41.228	256	0.929	1273	41.343
	5	280	0.996	1273	38.639	270	0.969	1273	38.774
**H15**	1					1140	0.225	1273	5.983
	2	1128	0.355	1273	10.18	1137	0.403	1273	8.696
	3	319	1.054	1273	41.95	320	1.069	1273	41.733
	4	298	0.986	1273	43.585	295	0.985	1273	42.909
	5	269	0.794	1273	41.168	266	0.79	1273	40.959
**H12**	1					1155	0.251	1273	2.539
	2	1137	0.384	1273	8.96	1134	0.409	1273	8.516
	3	257	1.093	1273	43.194	249	1.081	1273	43.047
	4	221	0.948	1273	40.112	220	0.96	1273	39.967

	5	252	0.733	1273	40.852	249	0.739	1273	40.834
**Delta**	1					1137	0.235	1271	2.586
	2	1134	0.372	1271	7.313	1130	0.428	1271	6.871
	3	264	0.975	1271	41.554	255	0.986	1271	41.312
	4	274	1.145	1271	43.263	270	1.142	1271	42.819
	5	163	0.877	1271	37.104	201	0.967	1271	37.125
**H8**	1					857	0.767	1271	9.662
	2	1132	0.558	1271	13.314	905	0.779	1271	10.35
	3	299	0.961	1271	40.472	263	0.988	1271	38.3
	4	283	0.997	1271	40.019	251	1.063	1271	36.872
	5	219	0.993	1271	40.903	95	1.195	1271	39.696
**H7**	1					930	0.769	1273	9.946
	2	1133	0.634	1273	13.186	863	0.762	1273	10.149
	3	345	0.96	1273	42.435	331	1.052	1273	40.286
	4	307	0.857	1273	37.405	336	1.053	1273	33.923
	5	318	0.984	1273	38.645	267	0.77	1273	37.045
**Alpha**	1					1136	0.264	1270	3.27
	2	1135	0.395	1270	8.603	1130	0.401	1270	8.373
	3	261	0.982	1270	41.407	256	1.009	1270	41.126
	4	244	0.915	1270	40.138	242	0.888	1270	40.296
	5	267	0.883	1270	38.076	251	0.768	1270	37.998
**H1**	1					1143	0.177	1273	4.112
	2	1133	0.398	1273	9.298	1138	0.407	1273	8.305
	3	268	1.036	1273	40.739	268	1.06	1273	41.654
	4	221	1.094	1273	41.279	170	1.157	1273	42.5
	5	281	1.044	1273	39.243	263	1.04	1273	39.897
**H3**	1					974	1.085	1270	14.345
	2	1098	0.497	1270	15.832	964	1.104	1270	8.161
	3	326	1.06	1270	48.098	288	1.036	1270	45.167
	4	300	0.966	1270	41.418	279	1.004	1270	37.025
	5	316	1.095	1270	45.807	293	1.134	1270	42.505
**H4**	1					921	0.759	1273	10.424
	2	1136	0.623	1273	13.479	864	0.749	1273	10.042
	3	334	1.025	1273	43.048	327	1.137	1273	40.702
	4	331	0.984	1273	37.786	307	1.067	1273	35.665
	5	278	1.083	1273	42.441	259	1.065	1273	42.115
**H5**	1					934	0.758	1273	10.095
	2	1139	0.617	1273	13.368	875	0.755	1273	10.099
	3	314	1.019	1273	41.719	301	1.081	1273	39.347
	4	281	0.853	1273	41.225	271	0.972	1273	40.211
	5	299	0.958	1273	37.739	253	0.856	1273	35.566
**Wuhan**	1								
	2	1140	0.394	1273	6.440				
	3	267	0.995	1273	40.109				
	4	186	1.187	1273	41.531				
	5	274	1.065	1273	39.842				
