# Supplementary material for: AlphaFold2 Reveals Structural Patterns of Seasonal Haplotype Diversification in SARS-CoV-2 Spike Protein Variants
Source: Biology (Basel). 2024 Feb 21;13(3):134. doi: 10.3390/biology13030134 (PMC10968544; doi:10.3390/biology13030134)
Supplement: Supplementary file 1 [file biology-13-00134-s001.zip › biology-2841665-supplementary/Supplementary/Table S3.pdf]

Table S3. Benchmarking results for three domain regions of the modeled Wuhan strain S-protein structure against each of three experimentally determined S-protein structures, which were identified using PDB accession codes. Three metrics are used to assess the quality of the modeled structure against the experimental references: Global Distance Test – Total Score (GDT-TS), Template Modeling Score (TM)(TM score/number of residues for L1 and L2), and Root Mean Square Deviation (RMSD)(Å/number of residues), with L1 and L2 representing the lengths of Wuhan WT segment or the length of the modeled segment, respectively.

	**Closed state: 6VXX-A**			**Open state: 6VYB-A**			**Open state: 6VYB-B**		
	**GDT-TS**	**TM**	**RMSD < 5 Å**	**GDT-TS**	**TM**	**RMSD < 5 Å**	**GDT-TS**	**TM**	**RMSD < 5 Å**
**NTD**	97.936	0.772/279<br>0.985/218	0.71/218	95.952	0.741/279<br>0.980/210	0.85/210	97.783	0.72050/279,<br>0.98701/203	0.62/203
**RBD**	93.063	0.832/223<br>0.956/193	1.01/189	93.878	0.853/223<br>0.958/198	0.95/193	81.18	0.70537/223<br>0.83950/186	0.90/156
**S2**	97.691	0.931/462<br>0.993/433	0.66/433	97.384	0.924/462<br>0.992/430	0.68/430	97.454	0.92770/462<br>0.99167/432	0.69/432
